# BioModels: Content, Features, Functionality, and Use

**DOI:** 10.1002/psp4.3

**Published:** 2015-02-26

**Authors:** N Juty, R Ali, M Glont, S Keating, N Rodriguez, MJ Swat, SM Wimalaratne, H Hermjakob, N Le Novère, C Laibe, V Chelliah

**Affiliations:** 1 European Bioinformatics Institute (EMBL-EBI), European Molecular Biology Laboratory, Wellcome Trust Genome CampusHinxton, Cambridge, UK; 2 Babraham Institute, Babraham Research CampusCambridge, UK.

## Abstract

BioModels is a reference repository hosting mathematical models that describe the dynamic interactions of biological components at various scales. The resource provides access to over 1,200 models described in literature and over 140,000 models automatically generated from pathway resources. Most model components are cross-linked with external resources to facilitate interoperability. A large proportion of models are manually curated to ensure reproducibility of simulation results. This tutorial presents BioModels' content, features, functionality, and usage.

## BACKGROUND

Mathematical models play an important role in the interpretation and investigation of mechanisms underlying complex biological systems, and thereby in the understanding of human diseases. Using the ever increasing amount of high quality quantitative data produced in the Life Sciences, and experimentally rooted mathematical models and simulations, it is now possible to validate our current understanding of biological processes and to generate meaningful hypotheses for ill-defined processes. This in turn drives further experimental design.

Mathematical modeling of biomolecular processes has become a standard part of the molecular and systems biologists' toolkits. Wide availability and interoperability of models is seen as hugely important in an increasingly collaborative field. However, models covering even the same domain can exhibit much variation since different scientists have developed them at different periods, coming from different perspectives and using different conventions. To increase the reusability of existing published models, there has been a significant drive toward standardization of model encoding, interoperability, distribution, and reuse.

A first step to encourage common development procedures in mathematical modeling was the definition and adoption of standard and machine-readable encoding formats. Efforts in this area include Systems Biology Markup Language (SBML),^1^ which has become the most widely used markup language in systems biology. As with other types of scientific research data, the next step was to provide freely accessible repositories of existing models. Such repositories fulfill several functions: models can be deposited to provide access to interested parties, can be directly retrieved for use, or can be used as a starting point for further improvements and refinements. In addition, such model repositories can be viewed as providing reusable components, from which submodels can be extracted to compose novel models, or to aggregate entire models into supermodels.

In order to promote interoperability between models and data, it is necessary to unambiguously identify all model components. One mechanism is to use standard cross-references^2^,^3^ to external resources that describe the nature of the model component as intended by the model encoder. This allows, for example, the specification of a model element as being a particular metabolite, protein, or gene. Such cross-references facilitate model query, comparison, and processing, such as for model enrichment or merging.

BioModels^4^ was created with many of these issues in mind. It is a repository for mathematical models describing biological, biochemical, or biomedical processes. It is easily accessible and allows researchers to re-use, analyze, and investigate the dynamic properties of the model system. While it currently uses SBML as its core model format, one can nevertheless retrieve models in many alternative model encoding formats.

This tutorial describes the domain content, access features, and use of BioModels. It is organized into three main sections as follows: (1) an overview of BioModels' content which provide the details and the generations of two groups of models, (a) models published in the literature and (b) Path2Models, hosted in the database; (2) a comprehensive guide to BioModels Web interface that describes the facilities available to access and retrieve models, functionalities available for each model, and additional services provided to facilitate the use of the repository by the community; and (3) a content usage section, illustrating how models are shared and re-used to answer different biological questions, and other general use of BioModels' content followed by conclusions. For easy navigation through the content of the tutorial, a structural overview is provided as a supplementary material SI-1.

## OVERVIEW OF BIOMODELS' CONTENT

The content of the database can be divided into two categories: a) models that have been described in the scientific literature, and b) models that have been generated through automated processing of pathway resources (Path2Models).^5^,^6^

The diversity of models within BioModels that are stored in SBML format illustrates that SBML provides a means of encoding models from a wide range of fields and disciplines within the broader biological context.^5^,^7^ Accepted as a *lingua franca* for exchange of such models, SBML is supported by a large number of software applications,^8^ providing a variety of simulation and analysis techniques, as well as enabling modelers to easily create or expand models without any need to be familiar with the underlying format.

SBML continues to be developed in stages, with a specification released at the end of each development cycle. Major editions of SBML are termed *Levels* and represent substantial changes to the composition and structure of the language. Within each given level, smaller refinements are made and released as new *Versions* of the given level. The latest level currently available is Level 3 (http://sbml.org/Documents/Specifications) which is being developed as a modular language, with a central core based on SBML Level 2, and extension packages layered on top of this core to provide additional, optional, sets of features. **Table** 1 illustrates the features supported by the different levels and versions of SBML. Although it is usually possible to convert from an older level/version combination to a later one, some of the features introduced in later levels/versions cannot be represented in earlier levels of the format.

**Table 1:** SBML components and their availability in different levels, versions, or packages

	Level 1	Level 2		Level 3	
	Versions 1–2	Version 1	Versions 2–4	Version 1	Package
Compartment	X	X	X	X	
Species	X	X	X	X	
Parameter	X	X	X	X	
UnitDefinition	X	X	X	X	
Rule	X	X	X	X	
Reaction	X	X	X	X	
Event		X	X	X	
Events with priority				X	
FunctionDefinition		X	X	X	
InitialAssignment			X	X	
Constraint			X	X	
CompartmentType			X		
SpeciesType			X		
QualitativeSpecies					Qual
Transition					Qual
SubModel					Comp
FluxBound					Fbc
Objective					Fbc
Layout					Layout

Model components that are used in SBML together with the SBML level, version, or package for which these components are available.

The next section describes the content of the database and the process leading to publication of models.

## MODELS PUBLISHED IN THE LITERATURE

The hosting of models described in literature within BioModels was launched in April 2005, and currently contains over 1,200 models described in approximately 160 different organisms. Most models in this category are kinetic models that describe and quantify interactions between biological components. Such models are subjected to extensive curation and annotation procedures.

### Model submission and provenance

Submission of models to BioModels is open to all users. Models can be submitted *via* an online interface and are accepted in two formats: SBML^1^ and CellML.^9^ On submission, each model is assigned a unique and stable submission identifier (of the form “MODEL” followed by a 10 digit timestamp), which allows users to access and retrieve it. Publication authors can use this identifier to reference their model in BioModels.

**Figure 1:**
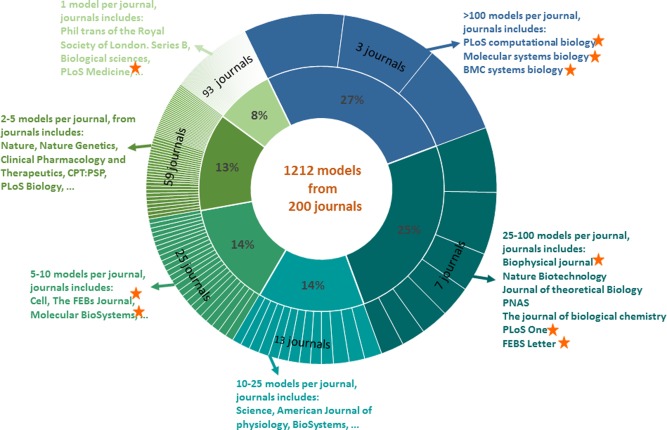
Source of literature models. As of September 2014, 1,212 models originate from 200 scientific journals. The inner layer of the chart gives the percentage of models that originate from journals listed in the second layer. For example, from the blue segment of the chart it should be understood that 27% of the models come from three journals (i.e., more than 100 models from each of the three journals). Approximately 50% (611 models) of the models are from 10 journals, of which 6 journals recommend model submission to BioModels in their authors' instructions (indicated by stars). This diagram not only illustrates the diversity of journals from which models are obtained, but also the importance of journal's support to the resource through encouraging authors to submit their models to BioModels, during the paper submission process.

Models published in the literature may either be encoded directly by curators or submitted by modelers and author(s) of the publications. Additionally, models were contributed to the resource through collaborations with other model repositories such as the former SBML model repository (California Institute of Technology, Pasadena, CA), JWS Online,^10^ the Database of Quantitative Cellular Signalling,^11^ and the CellML Repository.^12^ Several publishers of scientific journals recommend deposition of models to BioModels in their authors' submission guidelines. This includes journals from the EMBO press, Public Library of Science, Royal Society of Chemistry, BioMed Central, Elsevier, and FEBS Publishers. As a result, and due to the increasing awareness of BioModels within the community, the direct submission of models by authors/modellers is increasing rapidly. The total number of literature models reached 1,212 as of September 2014. These models originate from 200 scientific journals (**Figure** 1). Approximately 50% (611) of the models are from just 10 journals, of which 6 journals recommend model submission to BioModels in their authors' instructions.

A reference to the publication describing the model is always stored within the model file itself. It can be provided at the time of submission either as a PubMed Identifier, a digital object identifier, or as a uniform resource locator. While models may be submitted prior to publication of the associated paper, they are only made publicly available afterwards.

### Curation phase

**Figure 2:**
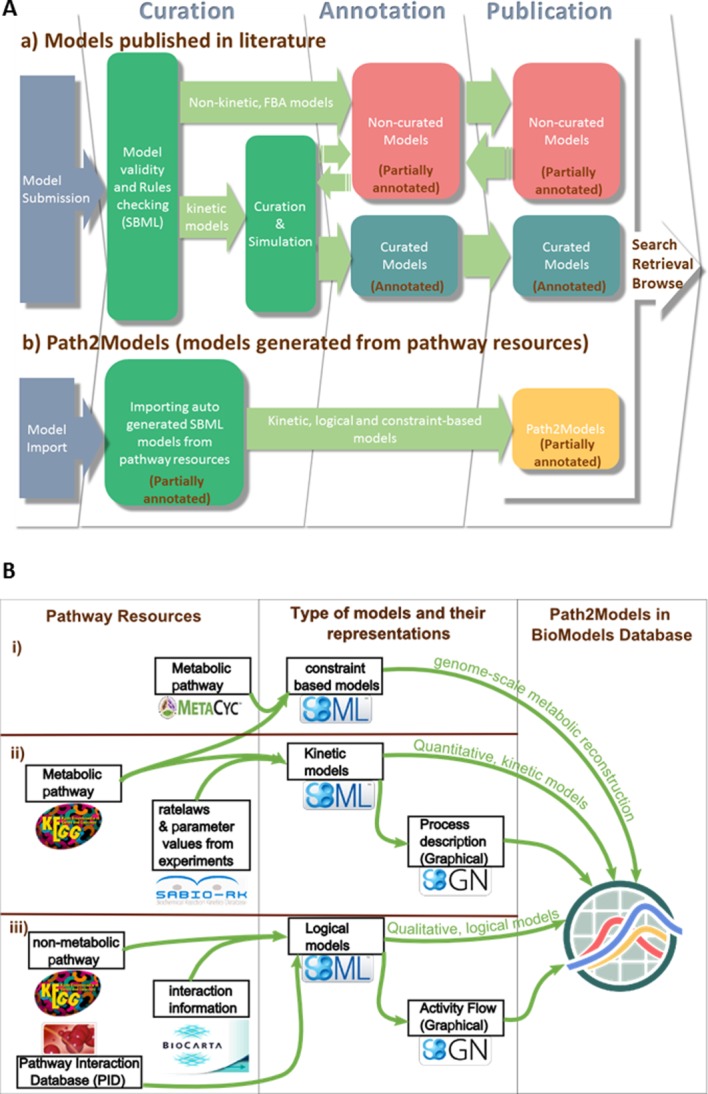
Model production pipeline of BioModels. (A) The diagram schematically represents the model generation pipeline of the two branches ((a) models published in literature and (b) Path2Models) of BioModels. The literature models undergo a sequence of steps from submission until publication in BioModels. Models imported from pathway resources are converted to SBML with additional information (see B for details), and are submitted to BioModels. (B) Detailed representation of the workflow of models generated from different pathway resources. Mathematical features, such as kinetic rate equations and flux bounds are added during the process, along with a graphical description.

Following submission, models pass sequentially through several steps in a curation procedure, which ends in their publication through BioModels (**Figure** 2**A(a)**). Most of the literature models are quantitative kinetic models, while some are qualitative models such as metabolic network, flux balance analysis, logical, and Petri-net based models.

- Models submitted in older Levels or Versions of SBML are converted to the latest Level and Version of SBML, unless such conversion would result in information loss or introduce inaccuracies to the model.
- SBML consistency checks are performed using libSBML.^13^ This includes checks for unit consistency and mathematical expressions, etc.

Models at this stage are fully SBML compliant but are then segregated into one of two branches based on the subsequent curation status. Generally, models that satisfy the MIRIAM (Minimum Information Required in the Annotation of Models) guidelines,^14^ progress to the curated branch. In particular, these guidelines require that models (1) be encoded in a standard format, (2) be clearly related to a single reference from a scientific journal, (3) correspond to the processes listed in the reference publication, and (4) reproduce the simulation results under the conditions described in the paper. Models that do not satisfy the MIRIAM guidelines are moved to the noncurated branch.

- A large proportion of the kinetic models (45%: 548 out of 1,212 models) are manually curated (fully MIRIAM compliant), a process that includes checking the behavior of the model when instantiated in a simulation. The dynamic behaviors are verified with a tool that is different from the one used in the original paper, thereby precluding tool-specific errors or hidden dependencies. The tools most commonly used for simulation checks are COmplex PAthway SImulator,^15^ the SBMLodeSolver,^16^ SBMLSimulator,^17^ or the facilities provided by the Systems Biology Workbench.^18^ A representative figure or table from the paper is reproduced and is made available with the model, together with a description of how it was obtained. If the simulation fails to reproduce the output present in the original publication, the authors are contacted to find the cause of discrepancies.
- The curated models are assigned a curation identifier (of the form “BIOMD” followed by a ten digit number), which reflects when the model was processed; for example, “BIOMD0000000488” denotes the 488^th^ successfully curated model. As with submission identifiers, the curation identifiers are unique and stable and can be used for search, retrieval, and referencing the model in BioModels. It should be noted that noncurated models can be accessed by their submission identifier; while curated models can additionally be identified through their curation identifier. A third identifier type is associated with models from the Path2Models project (see “Path2Models” below).
- About 55% of the literature models are in the noncurated branch, of which 38% are quantitative kinetic models, and the remaining 17% are nonkinetic models (highlighted with a network icon in the display interface). These include models of genome-scale metabolic networks, flux balance analysis, logical models, and Petri-net. Due to their intrinsic lack of kinetic information, these models cannot be validated with respect to their output and reside in the noncurated branch. Some kinetic models remain in this branch for other reasons: (1) Models that can be curated, but remain temporarily in this branch until curators are able to process them. (2) Models do not reproduce the results published in the paper; this may be due to errors in the implementation of the model or due to missing information in the publication. In such cases, authors are contacted for clarification, and models which can be corrected are migrated to the curated branch. (3) Models may be described in a publication, but contain some components that cannot be encoded in SBML.
- Models are given a meaningful name, in accordance with MIRIAM guidelines, using the biological process they address. The convention in BioModels follows the generic scheme “FirstAuthorLastNameYear – Topic.” For example, “Bianconi2012 – EGFR and IGF1R pathway in lung cancer” (BIOMD0000000427),^19^ and “Proctor2013 – Effect of Aβ immunization in Alzheimer's disease” (BIOMD0000000488).^20^

### Annotation phase

In addition to the requirements listed in “curation phase,” the MIRIAM guidelines^14^ also require that a model is provided with the contact information of the author(s), the date and time of model creation and last modification, a precise statement about model terms of distribution, as well as cross-referencing model components to external resources (reference databases and ontological records). This additional information is added to the model description at the first stage of the annotation process.

Different model encoders will routinely use different acronyms to represent a given entity, resulting in proliferation of acronyms for the same biological component. For instance, “Glucose” is often labeled “Glu,” “Glc,” “Glucose,” or even “G.” Such an ambiguous nomenclature must be resolved to permit the identification of equivalent entities across models and beyond. The cross-referencing (annotation) of model elements to records from external database resources precisely relates them to the corresponding biological processes or physical entities they represent. This facilitates efficient search and retrieval of the models from the database, and also helps in model comparison, merging and expansion with novel information.

Models in the curated branch are always annotated at the model level and at the level of variables. In some cases, annotations are also added to the parameters and mathematical expressions. Models in the noncurated branch are always annotated at the model level. They may additionally carry other annotations (at the level of physical entities, reactions, parameters, etc.) provided by the authors with the submitted models. These additional annotations may be unchecked by the curators.

Annotations are made using resolvable Identifiers.org Uniform Resource Identifiers.^2^ Over 50 external resources and ontologies are used for model annotation purposes. Some of the predominantly used resources include Gene Ontology (GO),^21^ Chemical Entities of Biological Interest (ChEBI),^22^ Human Disease Ontology,^23^ NCBI Taxonomy, Brenda Tissue Ontology,^24^ and UniProt.^25^ Any resource that is listed in the Identifiers.org Registry can potentially be used to annotate models.

Additionally, SBML controlled annotations use qualifiers (http://co.mbine.org/standards/qualifiers). These qualifiers define the relation between the external resource and the model component. More details on this annotation scheme can be found in section 6 of the SBML specifications (http://sbml.org/Documents/Specifications). In addition to the SBML supporting tools offering access to annotations, curators have access to a purpose-built annotation facility, within the BioModels Web interface.

Annotations for models from the Path2Models portion of BioModels (**Figure** 2**A(b)**) are handled differently. This is described below in the “Path2Models” section, together with the process that generates these models (**Figure** 2**B**).

### Model publication

Following the curation and annotation phases, models are ready to be published and a notification is sent to the submitter. Models then become publicly accessible through BioModels. All models available in BioModels are provided under the terms of the Creative Commons CC0, Public Domain Dedication. This means that the encoded models are available freely for use, modification, and distribution, to all users.

### Classification of models based on GO terms

**Figure 3:**
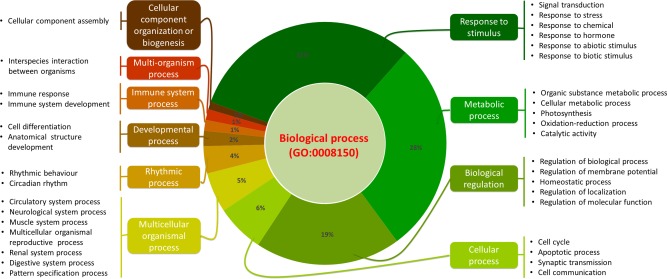
GO classification of models. All models are annotated with at least one GO term at the model level to represent the biological process it describes. In order to provide a broader level of classification of models based on these precise GO terms, three increasingly generic ancestor terms were chosen, the third term being one of the GO root term “Biological Process” (GO:0008150), which applies to all models. This type of hierarchical classification (from generic to most specific terms, through four GO terms) allows users to narrow down the search to specific biological process of interest. The top three GO terms used for the classification are shown, whereas the fourth term is the specific GO term used in the models. This allows categorized searching of models by following the GO term classification chart, and this facility is available through the BioModels website.

The annotations present in the models facilitate their efficient search and retrieval. All models are annotated with GO terms at the model level that represent, as accurately as possible, the biological processes that the model describes. In order to provide a broader level of classification of models based on these precise GO terms, three increasingly generic ancestor terms are chosen. In all cases, the third (broadest) term is chosen to be one of the GO root term “Biological Process” (GO:0008150), which applies to all models. This type of hierarchical classification (from generic to most specific terms, through four GO terms) allows users to narrow down their search to specific biological processes of interest. The three generic category terms are not stored in the model itself, but rather stored in the database. **Figure** 3 shows the GO classification of the models in both the curated and noncurated branch. The top three GO terms used for the classification are shown, whereas the fourth term is the specific GO term used in the models. For example, for BIOMD0000000488, the corresponding hierarchical classification begins with the most general GO term “biological processes” (GO:0008150), followed by “cellular component organization and biogenesis” (GO:0071840), “cellular component assembly” (GO:0022607), and ends with the most specific term “inclusion body assembly” (GO:00470841) that is used in the annotation of the model. This allows categorized searching of models by following the GO term classification chart, and this facility is available through the BioModels website.

### Record of model evolution

Wherever appropriate, literature models are cross-linked with the parent model(s) from which the structure and/or equations are derived or adapted. This information is stored in the SBML file using the qualifier “bqmodel:isDerivedFrom” and the identifiers from BioModels. Where BioModels identifiers are not available, for instance if the parent model has not yet been added to the database, the identifier for the publication (PubMed identifier or digital object identifier) in which the model is described is used.

**Figure 4:**
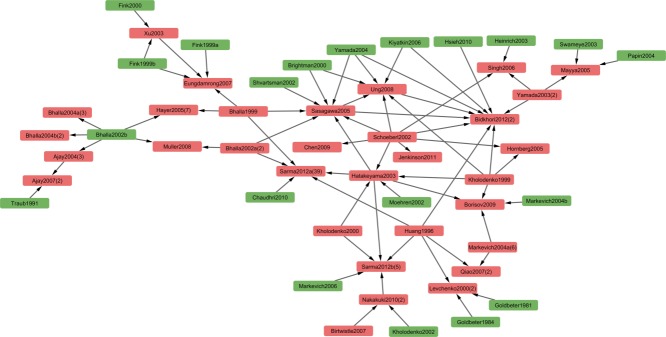
Importance of sharing and re-use of models. Clusters of models created based on the qualifier “bqmodel:isDerivedFrom” (see text for more information). The largest clusters are formed by models of signaling pathways. This diagram shows how signaling models are shared and re-used by different groups to develop more comprehensive models. The network is formed by the cross-talks of a range of signaling pathways activated downstream of ErbB, FGFR, NGFR, Insulin receptor, NMDAR, and AMPAR. The arrows (→) are used to link models that are derived/adopted from one or more parent models. Models available from BioModels are highlighted in salmon, otherwise in green. The models are named with the first author's surname followed by the year of publication. For some models, Author'sLastNameYear is followed by a number in brackets denoting the number of models in BioModels coming from the same publication. For example, “Bidkhori2012(2)” means that two variants of the model are described in the paper and both are available in BioModels. The two models “Bidkhori2012 - normal EGFR signalling” (BIOMD0000000452) and “Bidkhori2012 - EGFR signaling in NSCLC” (Bidkhori *et al*., 2012, BIOMD0000000453), describe the cross-talk mechanism between Ras/ERK, PI3K/AKT, and JAK/STAT pathways in normal and NSCLC non–small-cell lung cancer (NSCLC) cells, respectively. As can be seen from the figure, the model is itself derived from several other models.

This model lineage derivation shows currently over 150 clusters encompassing 819 models in total, with individual clusters ranging from 2 to over 90 models. A total of 561 of the models are already present in BioModels. **Figure** 4 shows the largest network of signaling pathway models. This network presents models describing multiple signaling pathways downstream a diverse range of receptor types, including ErbB, FGFR, NGFR, Insulin receptor, NMDAR, and AMPAR. This shows the importance of sharing and reuse of existing models to generate more elaborate models. For example, models “Bidkhori2012 - normal EGFR signaling” (BIOMD0000000452) and “Bidkhori2012 - EGFR signaling in NSCLC” (BIOMD0000000453), describe the cross-talk mechanism between Ras/ERK, PI3K/AKT, and JAK/STAT pathways in normal and non–small-cell lung cancer (NSCLC) cells,^26^ respectively, and are both derived from several other models.

## PATH2MODELS (MODELS DERIVED FROM PATHWAY RESOURCES)

The Path2Models^5^,^6^ section in BioModels provides computational models derived from pathway resources (**Figure** 2**A(b)**). The pathway information from data resources such as the Kyoto Encyclopedia of Genes and Genomics,^27^ BioCarta/Nature Pathway Interaction Database,^28^ MetaCyc,^29^ and SABIO-RK^30^ are automatically converted into standard-compliant computational models. Multiple biological processes, described in 2,616 organisms, were converted into computational models encoded in the SBML format, some of which are additionally available in SBGN-ML format.^31^ Depending upon the source data, three types of models have been generated: (i) Genome-scale metabolic reconstructions: these models are generated by extracting pathway data from the Kyoto Encyclopedia of Genes and Genomics and MetaCyc databases and subjected to flux balance analysis; (ii) quantitative, kinetic models of metabolic pathways: these models are generated based on the metabolic pathways distributed by the Kyoto Encyclopedia of Genes and Genomics and described as processes, in combination with experimentally determined rate laws and parameter values from the SABIO-RK database; (iii) qualitative, logical models of nonmetabolic (primarily signaling) pathways: these models are generated based on the nonmetabolic pathways distributed by the Kyoto Encyclopedia of Genes and Genomics and Pathway Interaction Database, with additional information from BioCarta pathways. **Figure** 2**B** illustrates the workflow used for the construction of the above mentioned three types of models from pathway resources.

All of these models are semantically annotated according to the MIRIAM guidelines: the model components (metabolites, genes, proteins, enzymes, reactions, etc.) are cross-linked with external data resources and controlled vocabularies, akin to the process for the literature based model, although assignments are made automatically based on the information present in the source data resources. A third type of BioModels identifier has been introduced to distinguish these automatically generated models from the literature models of the form “BMID” followed by 12 digits. The models in this branch are provided as ready-made models that can be used as initial starting models, where the kinetic data from experiments can be introduced for further simulation and analysis.

### Comprehensive guide to BioModels' web interface

Through its rich graphical user interface, BioModels provides a variety of methods to locate and access specific models, and to download them in a variety of formats. Model information is displayed in various ways, such as maps and textual reports. One can perform several analyses, for instance simulating the model and extract model components as submodels. Moreover, since each model is enriched with reference information drawn from diverse biologically relevant data resources, BioModels can be regarded as a Web portal for the modeling domain.

### Browse content

The BioModels front page provides the details of the models currently available. A generic browse feature allows to list models in each of the BioModels branches (curated, noncurated, and Path2Models).

Curated and noncurated models can also be browsed using the GO terms that are associated with individual models. This information is stored in the SBML file itself, and can be viewed in the interface from the “Model tab” (see “Model Display page” below). An interactive chart of GO terms gives access to increasingly more precise terms used in the annotations of models (see *Classification of models based on GO terms* for details). An alternative way is to search models using GO terms, where the information is displayed as a tree. The expandable branches highlight the total number of models available in each category as a parenthesized value. This feature operates using a condensed list of GO terms predetermined to be relevant to BioModels.

Models in the Path2Models branch are listed in categories, according to the type of model (metabolic, nonmetabolic, or whole genome metabolic models). Whole genome metabolic models are listed by species, while metabolic and nonmetabolic models are associated with particular GO terms, and subsequently divided into lists, ordered by species.

## SEARCH AND RETRIEVAL OF MODELS

Models can be retrieved using their assigned identifiers. However, since a user may not know or recall this identifier, BioModels also incorporates a search engine for locating specific models. A simple search can be launched from any page, with an advanced search facility also available. In the execution of a search, three main information sources are utilized. Used sequentially: (i) metadata about the model (high level model information, including authors and publication information); (ii) information contained in the model itself, e.g., present in the “notes” element of the SBML files; and (iii) cross-reference information present in the models (whenever possible the search query is expanded using additional information fetched from external data resources).

Postprocessing of search results is used to improve the quality of information provided to the user. For instance, taxonomical search queries are expanded to take into consideration the relationships between taxons; a search for “mammalia” will additionally retrieve models annotated with “Homo sapiens” and “Mus musculus,” since they are descendants of the original query term.

In the next sections, which demonstrate the various features of the resource, we use the example of a user who has launched a query for “Alzheimer's disease” and decided to further explore one of the models listed in the search results: “BIOMD0000000488 (Proctor2013 - Effect of Aβ immunization in Alzheimer's disease).”^20^

## MODEL DISPLAY PAGE

**Figure 5:**
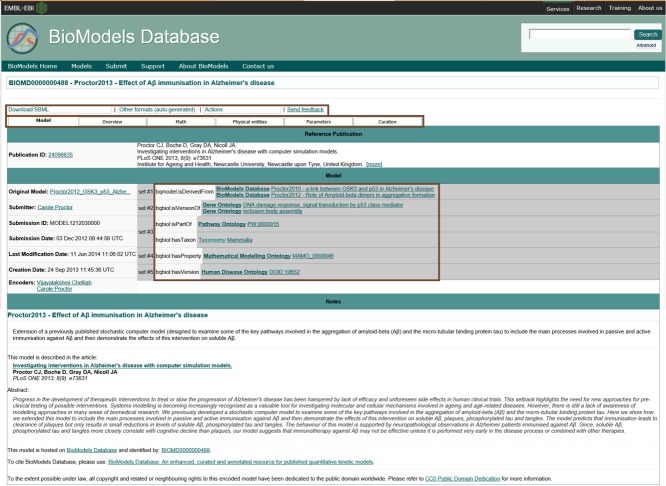
Screen image of a “Model” page. This displays the landing page of “Proctor2013 – Effect of Aβ immunization in Alzheimer's disease” (BIOMD0000000488). The page displays model download formats (Download SBML, Other formats (autogenerated)), links to features such as Online simulations (Actions), distribution of model components that span various tabs (Model, Overview, Math, Physical entities, Parameters, Curation), and model elements cross-linked (annotated) with various external resources such as Gene Ontology terms and Human Disease Ontology.

Once a model has been selected, either through browsing, or identified through search, a model page is displayed. Model components of manually curated models are organized, interlinked, and presented using tabs (**Figure** 5).

### Model tab

The default view when accessing a model, the *model tab* displays information about the model as a whole. It provides core information, such as the model name, publication information, link to the original file (original submission, either in SBML or an alternative format that is used as the basis to write the SBML), names of the encoder(s) and submitter, submission and modification dates, etc. In addition, this view also provides the most relevant GO terms (in this case “Inclusion body assembly”), reflecting the biological processes represented by the model, together with the taxonomic range (here “Mammalia”) to which the model may be relevant. The human readable notes provided by the submitters and curators are also displayed. In this case, the model is also annotated with specific disease information (Alzheimer's disease).

Upon further examination, it is apparent that this model (BIOMD0000000488)^20^ is derived from earlier versions BIOMD0000000286^32^ and BIOMD0000000462.^33^ The annotation states this relationship using the qualifier “bqmodel:isDerivedFrom.”

### Overview tab

This summary tab lists all the components of the model, whether they are physical entities, individual parameters, mathematical relationships between entities, or defined events. Each component is linked to a detailed view in the corresponding tab listed below. Selection of a component in the “Overview” acts as a shortcut, redirecting the user to a specific tab, where the selection made is highlighted, for convenience.

A user may wish to explore only part of a model, or use this part to create a submodel. Users can, therefore, select the components of interest. The other tabs subsequently only display the relevant parts of the model. For instance, if a molecular species is selected, only the reactions in which this species participates are displayed in the *math tab*. The user can then click the “Create a submodel with selected elements” link. This generates an additional tab in the viewing area, entitled “Submodel *n*,” where *n* denotes the n^th^ number of submodel that is created. The resulting submodel is the minimal valid SBML model that encompassed all the selected components and the relevant mathematical relationships. This model can then be downloaded.

### Math tab

The *math tab* lists the mathematical relationships linking model components. These mathematical constructs cover reactions, events, and explicit mathematical formulae (SBML rules). Each construct may be provided with further details, including any compartment restrictions, parameter value where applicable, a rendered mathematical equation, and optional cross-references to relevant Systems Biology Ontology (SBO) terms^3^ or GO terms.

### Physical entities tab

The *Physical entities tab* lists the physical or conceptual entities described in the model. Entities consist of the compartments and various entity pools (protein, chemical, etc.) that occupy specific compartments (cell, mitochondria, etc.). Thus a chemical species present in several compartments is considered as a set of different entities. Each entity may be listed with its initial size and cross-references to external databases, specifying the identity of the entity, and other annotations to specify the nature of the entity in the context of the model (protein complex, ligand, etc.).

### Parameters tab

The *Parameter tab* provides the list of every parameter used in mathematical relations. Parameters are grouped according to their scope (global or reaction specific), and where applicable, linked to the appropriate section of the Math tab. Parameter values for few models are also provided, potentially annotated with ontology terms to describe the type of parameter (dissociation rate constant, forward rate constant, etc.).

### Curation tab

The curation tab describes the result of the reproduction step of the curation process. It provides a representative plot generated by the curators reproducing one or more results present in the original paper. In addition to the typical graph, there may be some clarifications or amendments to the published procedure, which were found necessary to obtain correct simulation results. If changes are made to the model in order to reproduce the simulation figures, the changes are described and relevant additional files are provided. For an example, see the curation tab of the model “Lemaire2004 – Role of RANK-RANKL-OPG pathway in bone remodeling process” (BIOMD0000000278).^34^

### Model download

The models in BioModels are stored internally as SBML files that can be downloaded from each model page. Since the SBML standard has evolved over time, it is possible that the original submission file may be in a form that has been superseded. Since sequential SBML formats are largely backwards-compatible, it is possible to automatically convert a model between these alternatives. Users are, therefore, also able to download curated models in many alternative SBML Levels and Versions. The file used to generate the curation figure is highlighted.

Users can also download models in other formats. These alternative formats are produced from the SBML using converters developed for this purpose. This includes tool specific formats such as XPP,^35^ Octave (MatLab m-file; http://www.gnu.org/software/octave/), SciLab (http://www.scilab.org/), and VCML.^36^ In addition, there are converters to generate other standard formats, such as BioPAX (http://www.biopax.org/). We also provide a PDF report, providing human-readable summaries of model components and structure.^37^

In addition to individual model files, a bulk download of repository content is also available. Model archives are generated for each release and are provided *via* the EBI FTP server (http://ftp.ebi.ac.uk/pub/databases/biomodels/releases/). These provide access to only the SBML files of the literature models, all the model files (all formats in addition to the SBML files) of the literature models, all the RDF/XML files for the curated models, and all models from Path2Models (in three archives for the metabolic, nonmetabolic, and whole genome metabolism models). In addition to the full database releases, some of the archives are automatically generated on a weekly basis.

### Actions button

Each model page permits execution of a number of actions, for example displaying various views on the model, or the capacity to instantiate a simulation.

The reaction graph of the model network are displayed in SVG and PNG. This view is generally created automatically, directly from the SBML file. In some cases, the graph drawn automatically are improved manually.

Users can also launch simulations directly for all curated models on BioModels' infrastructure. This opens a popup window that permits the selection of the SBML species to be displayed in the results, the simulation time, and the time steps. Simulation jobs are sent to the EBI compute cluster, with a link given to retrieve the results. Both the numerical results from the simulation, and the graphical output are available to download. For a subset of curated models, simulation *via* JWS Online^10^ is available.

### User feedback

BioModels encourages feedback from its users, and provides a number of avenues for this purpose. A “Send feedback” link is present on each model display page to post problems with this specific model. Furthermore, the “Contact us” button in the main panel bar provides links for:

- Email support related to the services provided (Web services, user interface)
- Email support related to repository content (model error reporting, update requests)
- Discussion list (general modeling community discussions)

Archives of the messages are hosted on SourceForge for service and discussion lists. In addition, SourceForge also hosts a ticket system (http://sourceforge.net/projects/biomodels), where bug reports can be provided, and new features requested.

## MODEL OF THE MONTH

“Models of the Month” (http://www.ebi.ac.uk/biomodels-main/modelmonth) are short articles showcasing selected models from the curated branch of the repository. They include some introductory information on the subject of the model, placing it into biological and theoretical contexts, and discuss the results and significance of the model simulations presented. Such articles can be used for teaching purposes, and to make models more easily accessible for newcomers in the field of bio-mathematical modeling.

## PROGRAMMATIC ACCESS TO MODELS

### Web services

An alternative way to access BioModels information is through Web services.^38^ Details on the available services can be found at http://www.ebi.ac.uk/biomodels-main/webservices.

Using these Web Services, it is possible to retrieve extensive information from the model repository. The method “getModelSBMLByID” retrieves the SBML file of a given model using its unique identifier. This can be used, for instance, to get the “Alzheimer's disease” model using getModelSBMLByID(“BIOMD0000000488”). This method accepts both the submission (“MODEL”) and publication (“BIOMD”) forms of identifiers. If neither identifier is known to the user, it is also possible to use the methods “getModelsIdByName,” “getModelsIdByPerson,” and “getModelsIdByPublication” to retrieve a list of model identifiers when the model name, one of the authors, or a publication identifier (PubMed identifier or digital object identifier) are known. Furthermore, to assist users in the retrieval of models specific to a particular subject area, it is also possible to use the Web services to list models associated with a particular Gene Ontology term: the methods “getModelIdByGO” and “getModelIdByGOId” can be used to perform a query using a Gene Ontology term such as getModelIdByGO(“inclusion body assembly”), or the GO term identifier, getModelIdByGOId(“GO:0070841”). Similar methods are available for other types of resources. These services can also be used to generate a sub-model, given a model identifier, and specific SBML model component identifiers.

### SPARQL endpoint

BioModels SPARQL endpoint (http://www.ebi.ac.uk/rdf/services/biomodels/sparql) provides access to the content of BioModels as a linked dataset. This consists of an RDF representation of all curated models from the literature, comprising 174,156,735 triples, and 34,311,872 cross-references pointing to 2,781,565 different biological concepts (as of September 2014). It is part of a wider EBI effort to provide access to data using semantic Web technologies: the EBI RDF platform.^39^

Bioinformatics resources cross-reference each other and have direct links to ontological terms. These mappings can be used to construct queries which retrieve and integrate information from different linked data resources. For example, one can query for ChEMBL protein targets present in a model such as BIOMD0000000488 and obtain the drug compound that interacts with it, while no direct reference to ChEMBL is recorded in this model. This is achieved by writing one query which integrates data from BioModels and ChEMBL *via* the common cross references to UniProt proteins (see “One Example Use Case” and Supplementary material SI-2 for more details).

## MODEL FORMATS CONVERTERS

The Systems Biology Format Converter framework (SBFC; http://sourceforge.net/projects/sbfc/) provides a generic framework into which many format manipulation tools can be incorporated. The objective is to allow conversion of models between the different file formats used in Systems Biology, many of which are tool-specific. Many such converters have been created historically by different investigators. SBFC, implemented in Java and available as a standalone program, provides interfaces where new and pre-existing converters can be minimally modified, and plugged in. It also allows sequential “chaining” of individual converters to allow conversion between input/output combinations that were not previously possible. BioModels relies on SBFC to generate exports in alternative formats.

## CONTENT USAGE

### Building blocks for developing new models

#### One example use case

Models distributed by BioModels can serve multiple purposes, either as a body of knowledge on existing processes or as building blocks for further development. As new mechanistic details and experimental data become available, existing models can be extended/modified to incorporate these new hypotheses. The following use case describes components of various models that have been extended progressively to describe biological problems in a different context. Where the referenced model is available in BioModels, its accession number is provided along with the publication from which it was derived.

**Figure 6:**
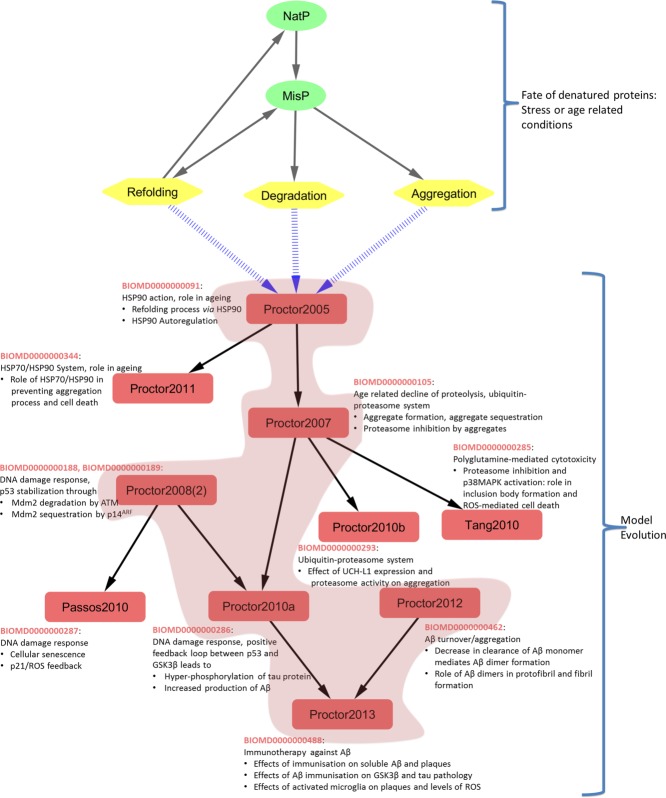
Evolution of BIOMD0000000488: Amyloid-beta (Aβ) immunization (Proctor *et al*., 2013). Under stress or age-related conditions, a correctly folded native protein (NatP) can become misfolded (MisP). There are three possible outcomes for misfolded proteins; the protein may be refolded into its native state with the help of chaperones such as HSP90, may be degraded and removed from the system, or may aggregate together. Such aggregates are the main characteristics of most neurodegenerative diseases, such as Alzheimer's disease and Parkinson's disease. The models in this figure describe to varying extents these three mechanisms. The arrows (→) are used to link models that are derived/adopted from one or more parent models. The models are named as described previously (Author'sLastNameYear) and numbers in parentheses denote the number of models in BioModels which result from the same publication. The mechanism described in each model is recorded adjacent to the model name along with its BioModels Identifier. The shaded portion in the figure depicts the model lineage for “Proctor2013” (BIOMD0000000488), which was built using components of previous models. Models in the unshaded regions do not contribute to the lineage of Proctor2013, but may be relevant for the lineage of other models. See text for further information. GSK3, glycogon synthase kinase 3; HSP, heat shock protein; ROS, reactive oxygen species.

The shaded region in **Figure** 6 illustrates the development history of the model BIOMD0000000488^20^ through various intermediate models from the same research group. This model (Proctor2013) includes the key processes involved in passive and active immunization against Amyloid-beta (Aβ) and demonstrates the immunological effect on Aβ and tau-protein aggregation, the major landmarks of Alzheimer's disease. The lineage of this model (**Figure** 6, shaded region) begins with BIOMD0000000091^40^ (Proctor2005), which describes the fate of misfolded proteins under stress and age-related conditions. It describes the role of Heat Shock Protein 90 and its autoregulation, while the degradation and aggregation processes are simplified in a single step. Molecular details on ubiquitin-mediated proteolysis and aggregation processes were incorporated in BIOMD0000000105^41^ (Proctor2007). Subsequent experimental studies suggested improvements to this model; glycogen synthase kinase 3 was shown to play a major role in Alzheimer's disease,^42^ with an additional potential role for p53.^43^ This link was explored in BIOMD0000000286^32^ (Proctor2010a), using components from BIOMD0000000105 (Proctor2007) and BIOMD0000000188^44^ (Proctor2008).

Predictions from this model (Proctor2010a) suggested, among other things, that stress mediated positive feedback between glycogen synthase kinase 3β and p53 led to an increase in Aβ production. Integrating this model with one that described the role of Aβ dimer levels in the initiation of the aggregation process BIOMD0000000462^33^ (Proctor2012), generated the model BIOMD0000000488^20^ (Proctor2013), which describes the effect of Aβ immunization in Alzheimer's disease. This model introduces a variable to represent Aβ immunization, and explores the effect of this intervention on soluble Aβ, plaques, etc.

Besides their contribution towards the evolution of BIOMD0000000488 (Proctor2013), components of BIOMD0000000091 (Proctor2005), BIOMD0000000105 (Proctor2007) and BIOMD0000000188/9 (Proctor2008(2)) are used as building blocks for developing various other models^45^–^48^ (unshaded region in **Figure** 6).

#### Possible extension using biomodels SPARQL endpoint search

There is an increasing number of research articles where the mechanism underlying the treatment (side-) effect, resistance to drug compounds, etc., are investigated using Systems Pharmacology modeling approaches (“Demin2013 – PKPD behavior - 5-Lipoxygenase inhibitors” (BIOMD0000000490),^49^ “Faratian2009 – Role of PTEN in resistance to trastuzumab” (BIOMD0000000424),^50^ Palmer *et al*.^51^), which provide new directions in the design of better therapies. A recent multiscale systems model-based analysis of interleukin-6 mediated immune regulation in Crohn's disease suggested that targeting dual components of the pathway is more effective than targeting a single component.^52^

One possible direction to understand the process of aggregation and the effect of treatment could follow these lines. BIOMD0000000488 predicted that the antibody against Aβ reduces the aggregation of both Aβ and tau, and that immunotherapy may not be effective unless it is performed at very early stages of disease progression, or combined with other therapies. Extrapolating these ideas, one way of extending this model would be to obtain the drugs for the protein molecules that are used in the model and study the effects of each drug or combinations of drugs on the aggregation process (disease progression). ChEMBL^53^ is a large-scale bioreactivity database, where one can use the bioactivity data to find associations between targets and drugs (which have been tested in the literature). BioModels SPARQL endpoint can be used to fetch the available drug compounds for the protein molecules used in the model. The query result may be filtered based on the half-maximal response concentration/potency/affinity and the current clinical development phase of the molecule (see Supplementary Materials S1 and S2, which are available online, for model details). Querying with varying combinations of these two parameters provides the potential target molecules in the model and their interacting compounds from ChEMBL.

### Advanced use cases

Besides its established, historical function as a central repository for mathematical models, BioModels is itself an input source for a variety of novel works. For example, models have been used as the input for workflows: qualitative models can, through a series of sequential operations involving Internet-accessible resources, be annotated to external resources, parameterized using experimental data, and processed to optimize assigned parameter values and generate results in a format-agnostic manner. Additionally, the semantic annotations can be used to identify components across which different models can be merged.^54^ Furthermore, it is also possible to analyze the individual model components, based on their semantic annotations, across the set of all models. This allows the clustering of related models (by shared components), and can be used to further assist in efficient search strategies.^55^ Semantic information can also be used to generate human-readable and queryable forms of curated models.^56^ BioModels content is also used to facilitate the standardized benchmarking of tools used for model simulations.

In conclusion, mechanistic models are becoming one of the cornerstones of computational life sciences. They allow us to understand the mechanisms underlying biological observations, make predictions, and test hypotheses. For example, modern drug discovery and design increasingly rely on mechanistic models. It offers insight into the nature of the complex PK and PD processes and offers a number of additional advantages, such as reduced extent of animal testing, and potentially increased accuracy of efficacy prediction and toxic effects. With the growing number and complexity of such models published in the scientific literature, it becomes necessary for these models to be deposited and preserved in a central repository in standard formats, and BioModels serves this purpose.

Models describing a wide range of biological processes at different biological scales are available through BioModels. Besides the usual curation tasks, efforts to collate, encode, and curate mechanistic models focusing on specific disease areas, are being undertaken. This has, for instance, resulted in the targeted curation and recent review on diabetes models.^57^

BioModels, with its vast selection of physiologically and pharmaceutically relevant mechanistic models, could play a prominent role in clinical domain. However, unlike models in Systems Biology (which are typically concerned with individual experimental data), for the drug discovery process, clinical trial results often manifest as population data which requires statistical modeling. One of the standard methodologies involves the use of nonlinear mixed effects modeling. Despite its statistical nature, at the very core of most nonlinear mixed effects models lies a deterministic prediction model, which in this context is called the “structural” model. The new XML based language, PharmML (Pharmacometrics Markup Language; http://www.ddmore.eu/pharmml), which is under active development by the DDMoRe consortium, provides the facility to encode the structural model with SBML. This will allow models from BioModels to be used for clinical data analysis.

So far, BioModels has mainly focused on models encoded in the SBML format, and the current software infrastructure is limited to SBML models. However, with the evolution of the modeling landscape, new challenges have arisen including the creation of new model formats to support specific domains. Therefore, following several queries from authors about submitting models in non-SBML formats, we are working to facilitate the hosting and distribution of non-SBML models from BioModels. Wherever possible, users are encouraged to submit models in COMBINE archive format (http://co.mbine.org/documents/archive). Moreover, an improved software infrastructure, JUMMP, being developed will allow the resource to widen its scope to contend with this issue in an effective manner. JUMMP is being developed collaboratively and is hosted on Bitbucket (https://bitbucket.org/jummp/jummp/).

Hosting models encoded in standard formats (such as SBML), the resource has grown substantially since its introduction in 2005. As of April 2014, it has expanded approximately 20-fold in content, with average model complexity rising fivefold over the same period (http://www.ebi.ac.uk/biomodels-main/static-pages.do?page=release_20140411).

The increasing number of journals describing models submitted to the resource (almost 200 journals) also indicates the growing awareness of BioModels by modelers in life sciences. The resource is accessed predominantly from Europe, North America, and Asia, serving over 65,000 unique visitors, with almost a million page views per year. In the past two years, content download from our FTP server has risen 10-fold.

BioModels, with its vast collection of biologically and biomedically relevant mechanistic models, serves as an invaluable resource for academic research, and for pharmaceutical and biotechnological industries.
