# Supplementary material for: BioModels: Content, Features, Functionality, and Use
Source: CPT Pharmacometrics Syst Pharmacol. 2015 Feb 26;4(2):e3. doi: 10.1002/psp4.3 (PMC4360671; doi:10.1002/psp4.3)
Supplement: Supplementary file 1 [file psp40004-00e3-sd1.docx]

**Supplementary Material S1 – Table of Content**

**BioModels Database: A guide to content, features,**

**functionality and use**

**Introduction**

**Background**

**Overview of BioModels’ content**

1. Models published in the literature

*Model submission and provenance*

*Curation phase*

*Annotation phase*

*Model publication*

*Classification of models based on GO terms*

*Record of model evolution*

1. Path2Models (models derived from pathway resources)

**A comprehensive guide to BioModels’ web interface**

Browse content

Search and retrieval of models

Model display page

*Model tab*

*Overview tab*

*Math tab*

*Physical entities tab*

*Parameters tab*

*Curation tab*

*Model download*

*Actions button*

*User feedback*

Model of the Month

Programmatic access to models

*Web services*

*SPARQL endpoint*

Model format converters

**Content Usage**

Building blocks for developing new models

*One example use case*

*Possible extension using BioModels SPARQL endpoint search*

Advanced use cases

**Conclusions**

**References**
