# Supplementary material for: BioModels: Content, Features, Functionality, and Use
Source: CPT Pharmacometrics Syst Pharmacol. 2015 Feb 26;4(2):e3. doi: 10.1002/psp4.3 (PMC4360671; doi:10.1002/psp4.3)
Supplement: Supplementary file 2 [file psp40004-00e3-sd2.docx]

**Supplementary Material S2**

**SPARQL endpoint search**

BioModels SPARQL endpoint (<http://www.ebi.ac.uk/rdf/services/biomodels/sparql>) provides an entry point to the content of BioModels database as a linked dataset. This consists of an RDF representation of all curated and non-curated models from the literature, comprising 174,156,735 triples, and 34,311,872 cross-references pointing to 2,781,565 different biological concepts (as of September 2014). It is part of a wider EBI effort to provide access to data using semantic web technologies: the EBI RDF platform[^1^](#_ENREF_1).

Bioinformatics resources cross reference each other and have direct links to ontological terms. These mappings can be used to construct queries which retrieve and integrates information from different linked data resources. For example, one can query for ChEMBL[^2^](#_ENREF_2) protein targets and the associated drugs for a particular model such as “Proctor2013 - Effect of Aβ immunisation in Alzheimer's disease” (BIOMD0000000488)[^3^](#_ENREF_3), while no direct reference to ChEMBL are recorded in this model. This is achieved by writing one query which integrates data from BioModels Database and ChEMBL via the common cross references to UniProt[^4^](#_ENREF_4) proteins (Figure S1).

In the query below, the *molecule_phase* corresponds to the current clinical development phase a compound is in. Once a compound has made it past phase 3, it is approved drug. Thus this query returns all approved drug compounds. The *pChembl* value allows a number of roughly comparable measures of half-maximal response concentration/potency/affinity to be compared on a negative logarithmic scale. For example, an IC50 measurement of 1nM would have a pChEMBL value of 9. pChEMBL is defined as: -Log(molar IC50, XC50, EC50, AC50, Ki, Kd or Potency).

PREFIX rdf: <http://www.w3.org/1999/02/22-rdf-syntax-ns#>

PREFIX rdfs: <http://www.w3.org/2000/01/rdf-schema#>

PREFIX owl: <http://www.w3.org/2002/07/owl#>

PREFIX xsd: <http://www.w3.org/2001/XMLSchema#>

PREFIX dc: <http://purl.org/dc/elements/1.1/>

PREFIX dcterms: <http://purl.org/dc/terms/>

PREFIX foaf: <http://xmlns.com/foaf/0.1/>

PREFIX skos: <http://www.w3.org/2004/02/skos/core#>

PREFIX sbmlrdf: <http://identifiers.org/biomodels.vocabulary#>

PREFIX cco: <http://rdf.ebi.ac.uk/terms/chembl#>

SELECT DISTINCT ?element ?annotation ?targetName ?moleculeName WHERE {

<http://identifiers.org/biomodels.db/BIOMD0000000488> ?p ?element .

?p rdfs:subPropertyOf sbmlrdf:sbmlElement .

?element ?qualifier ?idorgAnnot .

?qualifier rdfs:subPropertyOf sbmlrdf:sbmlAnnotation .

?idorgAnnot owl:sameAs ?annotation .

SERVICE <http://www.ebi.ac.uk/rdf/services/chembl/sparql> {

?targetcmpt cco:targetCmptXref ?annotation .

?target cco:hasTargetComponent ?targetcmpt ;

cco:hasAssay ?assay .

?target rdfs:label ?targetName .

?assay cco:hasActivity ?activity .

?activity cco:hasMolecule ?molecule ;

cco:pChembl ?molecule_pchembl .

?molecule cco:highestDevelopmentPhase ?molecule_phase .

FILTER(?molecule_pchembl > 6 )

FILTER(?molecule_phase = 4 ) .

?molecule rdfs:label ?moleculeName

}

}


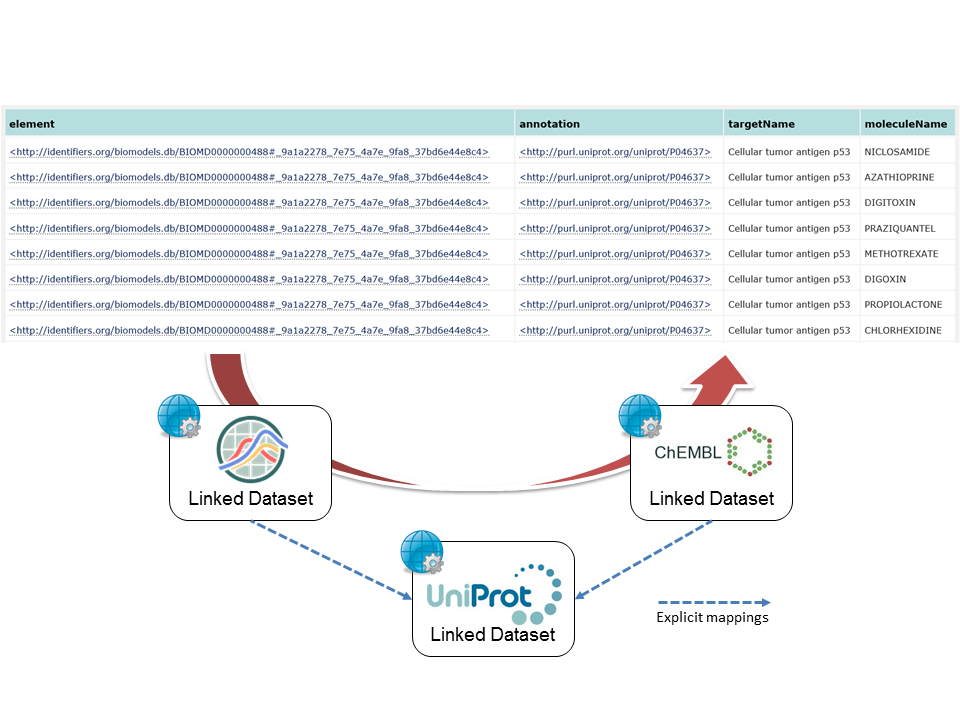


Figure S1: **BioModels SPARQL endpoint query for BIOMD0000000488.** BioModels Database is a repository of peer-reviewed biological models, while ChEMBL provides information on drugs and their targets. There exist no direct cross-references between these two resources. However, Linked Datasets of the two resources can be queried in a federated system, to discover relationships through a variety of third party linked datasets including UniProt. SPARQL enables such federated cross-resource query. Here, a query is used to discover which proteins represented in BIOMD0000000488 may be targets for drugs registered in the ChEMBML database. The results show the relationship between model components (‘element’), which are representative of proteins within the model, and are cross-referenced using UniProt (‘annotation’), and for which ChEMBL has registered an approved drug (‘moleculeName’).

Without BioModels Linked Dataset, one would have to write a small program which would call many different web services to achieve this result. The above query and other example queries are provided at <http://www.ebi.ac.uk/rdf/documentation/biomodels/queries>.

**References**

1. Jupp S, Malone J, Bolleman J, Brandizi M, Davies M, Garcia L*, et al.* The EBI RDF platform: linked open data for the life sciences. *Bioinformatics* 2014, **30**(9)**:** 1338-1339.

2. Bento AP, Gaulton A, Hersey A, Bellis LJ, Chambers J, Davies M*, et al.* The ChEMBL bioactivity database: an update. *Nucleic acids research* 2014, **42**(Database issue)**:** D1083-1090.

3. Proctor CJ, Boche D, Gray DA, Nicoll JA. Investigating interventions in Alzheimer's disease with computer simulation models. *PloS one* 2013, **8**(9)**:** e73631.

4. Apweiler R, Bairoch A, Wu CH, Barker WC, Boeckmann B, Ferro S*, et al.* UniProt: the Universal Protein knowledgebase. *Nucleic acids research* 2004, **32**(Database issue)**:** D115-119.
